# Indoor feeding combined with restricted grazing time improves body health, slaughter performance, and meat quality in Huang-huai sheep

**DOI:** 10.5713/ab.23.0252

**Published:** 2023-10-18

**Authors:** Yafeng Huang, Mengyu Zhao, Xiaoan Zhang, Huiqing Wei, Lumeng Liu, Zijun Zhang, Xiao Cheng, Guanjun Wang, Chunhuan Ren

**Affiliations:** 1 College of Animal Science and Technology, Anhui Agricultural University, Hefei 230036, China; 2 Center of Agriculture Technology Cooperation and Promotion of Dingyuan county, Dingyuan 233200, China; 3 Yingshang Agricultural Green Development Promotion Center, Yingshang 236200, China

**Keywords:** Body Health, Huang-huai Sheep, Meat Quality, Restricted Grazing, Slaughter Characteristics

## Abstract

**Objective:**

The aim of this study was to evaluate the effects of three feeding systems, i.e., indoor feeding (CON), indoor feeding with 4-h daily access to grazing artificial pasture (ITGP), and indoor feeding with 8-h daily access to grazing artificial pasture (IEGP), on the plasma antioxidant and immunological capacity, slaughter characteristics, meat quality and economic efficiency of Huang-huai lambs.

**Methods:**

Thirty-three healthy Huang-huai rams with similar body weight (approximately 5 mo of age, 28.96±1.01 kg) were assigned equally to three experimental groups. When finished fattening, six lambs from each group were collect blood samples for plasma analyses and then slaughtered to determine slaughter characteristics and obtain *biceps brachii* muscle for further analysis of meat quality and fatty acid profile.

**Results:**

Compared to CON group, animals submitted to ITGP and IEGP groups resulted in greater contents of serum glutathione peroxidase, immunoglobulins (IgA, IgG, and IgM), polyunsaturated fatty acids (PUFA), n-6 PUFA, and PUFA/saturated fatty acid (FA) ratio and lower palmitic/oleic acid ratio (p<0.05). Moreover, animals in ITGP group exhibited a higher (p<0.05) loin eye area, content of meat crude protein (CP), and eicosetrienoic acid compared to CON group, while slaughter performance was superior (p<0.05) to that of the IEGP group. The economic efficiency of ITGP group was 70.12% higher than that of CON group, while the IEGP group exhibited a decrease of 92.54% in economic efficiency compared to the CON group.

**Conclusion:**

Restricted grazing time combined with indoor feeding was more effective in conferring superior body health, carcass traits and economic efficiency in Huang-huai lambs, as well as higher CP content and healthier FA composition in the resulting meat.

## INTRODUCTION

Huang-huai sheep is a new breed that was developed in China in 2020. This breed exhibits characteristics such as high reproduction rate, fast growth speed, tolerance to roughage, and good meat quality [1], making it suitable for meeting the high-yield and the high-quality meat demands of today’s consumers. Therefore, enhancing the carcass performance and meat quality of Huang-huai sheep is essential for the development of high-quality animal husbandry and the mutton industry.

Different feeding systems have gained significant attention due to their association with beneficial effects on production traits, lambs’ health status, and consumers’ expectations for the intrinsic and extrinsic characteristics of lamb meat [2]. Consumers tend to prefer mutton produced on grazing pasture, which constitutes a healthier choice compared to mutton obtained from an indoor system [3]. However, it is important to acknowledge that overgrazing leading to ecological degradation has become a significant problem globally. Additionally, with the implementation of policies aiming to protect grassland ecosystems in China, the feeding systems of sheep have shifted from traditional grazing to pasture grazing combined with indoor fattening, or even full indoor fattening system [3,4]. Under the indoor feeding conditions, the activity of antioxidant enzymes and immunity tends to decrease due to the lack of physical exercise and the prohibition of antibiotics in animal feed, which further impacts animal health, production performance and mutton quality [5,6]. Therefore, there is a growing focus on finding suitable feeding systems that ensure maximum animal production without compromising the quality of the final product or increasing environmental pressure. Interestingly, the strategy of restricted grazing time combined with indoor feeding has been demonstrated as a more effective method for managing lamb production, improving growth performance, and maintaining high grassland productivity [7,8]. However, little is known about the impact of time-restricted grazing on artificial grassland and increasing levels of supplementation with indoor feeding diets on body health, slaughter performance, and meat quality of Huang-huai sheep.

Therefore, the aim of this study was to evaluate the effects of different feeding systems, i.e., indoor feeding, indoor feeding combined with daily four-hour grazing on artificial pasture, and indoor feeding combined with daily eight-hour grazing on artificial pasture, on plasma antioxidant and immunity capacities, slaughter traits, meat quality and economic efficiency of Huang-huai lambs. The results discussed herein provide valuable insights into novel ways of improving grazing management and supporting the sustainable and healthy development of the Huang-huai sheep industry.

## MATERIALS AND METHODS

The present experiment was conducted at the Huangzhu Animal Husbandry Co., Ltd (32°57′ N, 117°50′ E, 71.7 m average above sea level) from April to July in 2021. All animals handling procedures were approved by the Animal Care and Ethics Committee of the Anhui Agricultural University, China, in accordance with the respective institutional animal research guidelines (permit number SYXK 2016-007).

### Animals and experimental design

Thirty-three healthy Huang-huai rams with similar body weight (approximately 5 mo of age, 28.96±1.01 kg) were randomly chosen and assigned to three experimental groups. The three groups were: i) indoor feeding with no access to pasture (CON); ii) indoor feeding combined with time-limited (4 h) daily access to grazing on artificial pasture (ITGP); and iii) indoor feeding combined with 8-h per day access to artificial grazing pasture (IEGP). Animals in the ITGP group were allowed to graze from 9:00 to 13:00 during the initial 45 d of the adaptation period plus the days of the fattening period, and then grazed from 14:00 to 18:00 during the fattening period. Animals in CON and ITGP groups were fed the indoor diet *ad libitum* twice daily at 8:30 and 18:30 h, whereas animals in the IEGP group were fed the indoor diet *ad libitum* once daily at 18:30 h. Lambs in ITGP and IEGP groups grazed on artificial mixed grassland composed of alfalfa (*Medicago sativa*), white clover (*Trifolium repens*), orchard grass (*Dactylis glomerata*), perennial ryegrass(*Lolium perenne* L.) and other pasture plants. The experimental period lasted for 100 d and was preceded by a 12-d adaptation period. The supplementary ingredients and nutrient contents of diets are shown in Table 1. The amounts of supplementary diets offered were recorded daily, and the orts were weighed each day to estimate dry matter intake (DMI).

### Blood sampling and plasma analyses

Prior to the morning feeding on the final day of the feeding experiment, blood samples were taken from the jugular vein of six individuals within each experimental group in tubes without anticoagulant. Then, blood samples were clotted at room temperature for 30 min and centrifuged (3,000 g, 4°C and 10 min) to obtain plasma. Plasma samples were stored at −80°C until further analysis. The levels of immunoglobulin A (IgA), IgG, IgM and glutathione peroxidase (GSH-Px) were determined using an enzyme-linked immunosorbent assay kits following the manufacturer’s instructions (Shanghai Jianglai Biotechnology Co., Ltd., Shanghai, China).

### Animal slaughter, carcass characteristics and non-carcass components

At the finishing day of the feeding experiment, six lambs from each experimental group were randomly selected and individually weighed to determine slaughter live weight (SLW) after fasting for 24 h and fasting from drinking for 8 h, and then slaughtered by slaughtering professionals using the Halal method. Hot carcass weight (HCW) was determined immediately after slaughter. Dressing percentage (DP) was calculated by dividing the HCW by the SLW [3]. Non-carcass components including heart, kidney, liver, spleen, lungs, empty gastrointestinal tract (EGT) were separated and weighed to calculate the percentage of SLW. Moreover, subcutaneous fat, kidney fat, tail fat, and omental and mesenteric fat were weighed to calculate the percentage of HCW [9]. The loin-eye outline between the 12th and 13th ribs in each carcass was drawn on tracing paper, and the loin eye area (LEA) was measured using a scaled grid paper. The grade rule (GR) value (at 11 cm away from the middle over the 12th rib) represented the carcass fat content and was directly measured on all carcasses using a Vernier caliper [10]. The *biceps brachii* (BB) muscle was sampled from the right carcass side and frozen at −80°C for further analysis of chemical and fatty acid (FA) composition.

### Determination of meat quality parameters

The BB muscle was used to determine meat edible quality traits, i.e., pH, color and drip loss (DL). At 45 min and 24 h postmortem, meat pH was determined in triplicates using a portable pH-meter (FE20; Mettler Toledo International Inc., Zurich, Switzerland) at a depth of 2 cm into the meat sample. Meat color was measured with a Minolta chromameter (ADCI-WSI; Beijing Chentaike instrument Co., Ltd., Beijing, China) after a 30-min blooming period and expressed based on CIELAB color space parameters lightness (L*), redness (a*), and yellowness (b*). Color saturation (C*) was calculated as (a*^2^+b*^2^)^1/2^, whereas hue angle (H*) was calculated as tan^−1^ (b*/a*)×(180/π) [11]. Three independent measurements were conducted for each sample, and average was obtained. The DL was calculated as a percentage of the amount of water loss in meat samples after storage in a refrigerator at 4°C for 24 h to the initial weight of the samples (1 cm×1 cm ×2 cm) [12].

### Determination of meat chemical composition

Chemical analysis of meat samples was conducted in accordance with the methods prescribed by the Association of Official Analytical Chemists [13]. Crude protein (CP) analysis was performed using the Kjeldahl method, in which total nitrogen was multiplied by a conversion factor of 6.25. The intramuscular fat (IMF) content was determined by the Soxhlet method. Moisture content was determined by gravimetry in an oven at 105°C. Ash content was determined using a muffle furnace at an average temperature of 600°C.

### Determination of meat fatty acid content

The FA content of meat was analyzed as described by Chen et al [14]. Approximately 1.50 g of meat samples was initially solubilized into the mobile phase (2.65 mL of a solution consisting of 1 mL of hexane and 0.66 mL of C11:0 [1 mg/mL], 0.66 mL of ethyl alcohol [95%, *v/v*], and 1.33 mL of purified water). Then all samples were ground at 60 Hz for 2 min in a fully automatic sample rapid grinder. Subsequently, 33 mg of pyrogallic acid, 33 mg of zeolite, and 3.3 mL of hydrochloric acid (8.3 mol/L) were added to the sample, followed by incubation at 75°C in a water bath for 40 min. Then, 5 mL of 95% ethanol solution and 10 mL of ether and petroleum ether mixture (1:1; *v/v*) were added to the sample. The mixture was vortexed for 5 min, transferred to a separator funnel, and left to stand for 5 min to collect the filtrate; these steps were repeated thrice. The filtrate was then transferred to a rotary evaporator to concentrate until dry. Subsequently, 2% NaOH-methanol solution (1.6 mL) was added to the extract and followed by incubation for 3 min at 80°C in a water bath. Then, 1.4 mL of 15% boron trifluoride methanol solution was added, followed by incubation for 3 min at 80°C in a water bath. Then, after the flasks were left to cool to room temperature, and 2 mL of n-heptane was added, homogenized for 5 min, and then 3 mL of saturated aqueous sodium chloride solution was added and left to stand for 5 min. Then, 1.5 mL of the upper layer of the n-heptane extract was transferred into a centrifuge tube containing 0.6 g of anhydrous sodium sulfate, homogenized for 1 min, and left to stand for 5 min, then 1 mL of the supernatant was aspirated. The organic phase was separated and filtered through a 0.22-μm pore size filter membrane.

The FAs in samples were quantified using a gas chromatography (GC) (Agilent, 7890A; Agilent Technologies, Santa Clara, CA, USA) equipped with a DB-WAX capillary column (30.0 m×0.25 mm×0.25 mm) and a flame ionization detector. The GC temperature program was initially set to 60°C for 2 min, then increased to 200°C at a rate of 15°C/min; then increased to 230°C and maintained for 19 min at a rate of 3°C/min. The injector temperature was maintained at 240°C, whereas the volume of injected sample was 1 μL; nitrogen was used as carrier gas at a constant flow of 0.8 mL/min. The identification of FAs was performed using the standards preconized by Sigma-Aldrich (Deisenhofen, Germany). The contents of FAs in samples were calculated using chromatogram peak areas and expressed as mg/100 g of fresh meat in each sample.

### Economic efficiency analysis

The economic analysis of carcass production was determined by the cost of feed input and the price of carcass. The feed cost determined by considering the prices of raw materials used in supplementary diets and rental fees for grazing grassland during the experimental period. The total feed cost does not include basic expenses such as labor, equipment, electricity, and water. The carcass price was established based on the weight of lamb carcasses.

### Statistical analysis

The general linear model routine in SPSS software (Version 25.0. IBM Corporation, Armonk, NY, USA) was used to analyze the plasma antioxidant and immune indexes, slaughter performance and meat quality attributes by the one way analysis of variance. The effect of the three feeding systems on all variables was analyzed according the statistical linear model: Y_i_ = μ+M_i_+R, where Y_i_ is the dependent variable, μ is mean vale, M_i_ means the fixed effect of feeding system, and R means the random effect. The means were determined using Duncan’s test to determine the significance of differences at p≤0.05 and trends at 0.05<p≤0.10.

## RESULTS

### Plasma antioxidant and immunity capacities

Table 2 depicts the results of the impact of different feeding systems on plasma antioxidant and immunity capacities of Huang-huai sheep. Lambs in the CON group exhibited lower (p<0.001) plasma contents of GSH-Px, IgA, IgG, and IgM compared to the lambs in the other treatment groups. However, the plasma contents of GSH-Px, IgA, IgG, and IgM were similar between IEGP and ITGP groups.

### Slaughter performance

As shown in Table 3, the DMI value was lower (p<0.001) in IEGP group than in CON or ITGP group, with no difference between CON and ITGP group. In terms of carcass traits, the SLW, HCW, meat weight, and yield were higher in the ITGP group compared to the IEGP group, with the CON group having intermediate values (p<0.05). The values of DP (p = 0.05) and LEA (p<0.05) in the IEGP group were higher (p = 0.05) compared to the other two experimental groups. Moreover, bone weight and yield, meat/bone and GR were not influenced by the feeding systems, with average values of 5.93±0.031 kg, 12.44%±0.53%, 3.00±0.14, and 4.27±0.33 mm, respectively.

Furthermore, no differences were found among the feeding systems for non-carcass components, except for EGT. The average values for heart, liver, spleen, lungs, kidney and pancreas were 0.41%±0.01%, 2.01%±0.11%, 0.34%±0.06%, 1.03%±0.05%, 0.29%±0.01% and 0.10%±0.01%, respectively. The EGT value was lower (p = 0.05) in the CON group compared to the IEGP or ITGP groups, with no difference between the IEGP and ITGP groups. Considering body fat deposition, no differences for body fat deposition among three feeding systems, except for subcutaneous fat, which increased in the CON group compared to the IEGP and ITGP groups (p<0.05). Moreover, omental and mesenteric fat, kidney fat and tail fat averaged 3.41%±0.40%, 0.78±0.10 and 0.68%± 0.09%, respectively.

### Edible quality attributes

The pH_45 min_ and pH_24 h_ values of meat were similar among the different feeding systems, with average values of 6.49± 0.07 and 6.18±0.06, respectively (Table 4). The meat color in lambs submitted to the CON and ITGP groups presented higher L* values compared to the IEGP group. No differences were found for a*, b*, C*, H* and DL values, with average value of 18.78±0.91, 13.80±0.65, 23.44±0.94, 36.57±1.45, and 2.12±0.11, respectively.

### Chemical composition attributes

**Figure 1:**
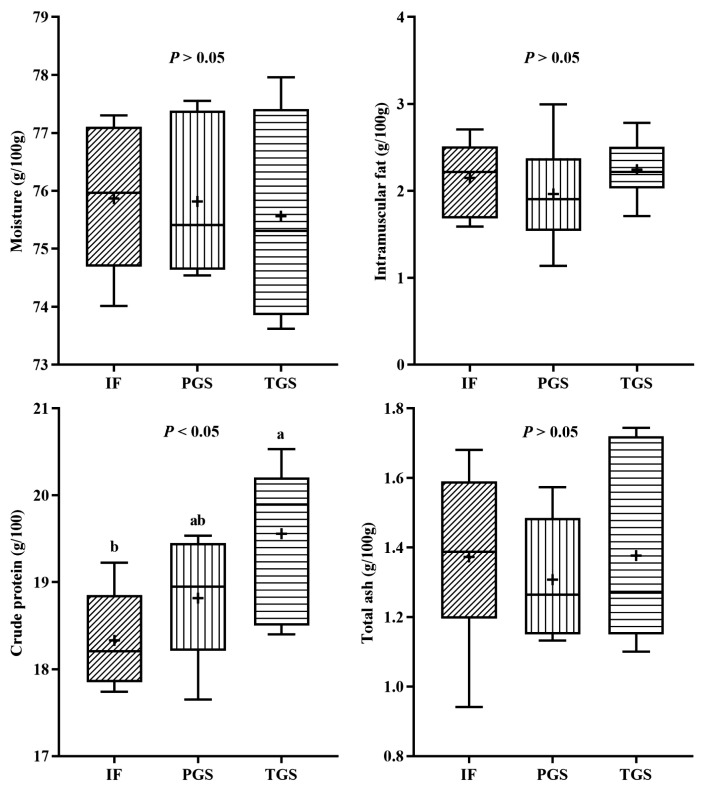
Effects of different feeding systems on the chemical composition in the *biceps brachii* muscle of Huang-huai lambs. CONT, indoor feeding with no access to pasture. ITGP, indoor feeding combined with time-limited (4 h) daily access to grazing on artificial pasture. ITGP, indoor feeding combined with 8-h per day access to artificial grazing pasture. +, average value. ^a,b^ Means with different letters differ at p<0.05.

The moisture, IMF and total ash contents of meat were not influenced by the feeding systems, with average values of 75.75%±0.32%, 2.12%±0.11%, and 1.35%±0.05%, respectively (Figure 1). The CP content in meat was lower in the CON group than in the ITGP group, with the IEGP group showing intermediate levels (p = 0.036).

### Fatty acid composition attributes

Among saturated FA (SFA), the content of heptadecanoic acid (C17:0) in meat was higher (p = 0.029) in the ITGP group, intermediate in the CON group, and lower in the IEGP group. Other individual SFA as well as the total SFA were not affected by the feeding systems (Table 5). Among monounsaturated FA (MUFA), the IEGP group exhibited a higher content of erucic acid (C22:1n-9) compared to the CON group, with the ITGP group being intermediate (p = 0.014). Other individual MUFA and total MUFA contents were similar among all feeding systems. Among PUFA, linoleic (C18:2n6) and arachidonic (C20:4n6) acid contents were higher in the CON group than those in the IEGP and ITGP groups (p<0.05), with no difference between IEGP and ITGP groups. Other individual SFA contents were similar among all groups, whereas an upward trend for eicosetrienoic (C20:3n6; p = 0.088) and eicosedienoic (p = 0.074) acid contents was observed in the ITGP group. The contents of total polyunsaturated FA (PUFA) (p = 0.003), n-6 PUFA (p = 0.002), PUFA/SFA (p = 0.004) were higher in IEGP or ITGP groups compared to the CON group, while the palmitic/oleic acid ration (C16:0/C18:1; p = 0.025) was higher in the CON group compared to IEGP or ITGP groups. However, these parameters in the IEGP group were not different from those in the ITGP group. The contents of n-3PUFA, and n-6/n-3 PUFA were not affected by the feeding systems.

### Economic efficiency

Compared to the CON group, daily feed costs were increased by 23.8% and 23.7% in IEGP and ITGP groups, respectively (Table 6). The carcass price of ITGP group was 17.32% higher than that of CON group, whereas the IEGP group exhibited a decrease of 13.61% in carcass price compared to the CON group. The economic efficiency of the IEGP group was lower by 92.54% than that of the CON group; however, it was increased by 70.12% for the ITGP group compared to the CON group.

## DISCUSSION

### Plasma antioxidant and immunity capacities

Oxidative stress is likely a primary factor contributing to the incidence of various diseases of ruminants [15]. The main parameters used for assessing the oxidative status of animals include determining GSH-Px content, in which increased levels of GSH-Px are advantageous for mitigating damage caused by peroxides and free radicals to the organism [15]. The present experiment indicated that animals submitted to IEGP and ITGP feeding systems led to a significant increase in GSH-Px levels by 30.51% to 35.27% in Huang-huai sheep. This suggests that the combination with grazing in addition to indoor feeding may increase the antioxidant capacity of Huang-huai sheep. This increase in animals of IEGP and ITGP groups associated with enhanced antioxidant capacity can be attributed to the higher physical activity and the presence of alfalfa in grazing pastures. Previous studies have also reported similar findings, demonstrating that dietary supplementation with alfalfa can increase the activity of GSH-Px in sheep serum [5].

Moreover, molecules involved in humoral immunity such as IgA, IgG, and IgM, reflect bodily immune functions [16,17]. In this study, the plasma content of IgA, IgG, and IgM was significantly increased in IEGP and ITGP groups. Although limited research has been conducted on ruminants regarding the response to different feeding systems, studies in monogastric animals such as pigs [16] and chickens [17] have shown that grazing systems have a positive effect on improving bodily health status. Collectively, the findings of this study support these observations, suggesting that grazing in the form of IEGP and ITGP feeding systems can improve the antioxidative and antibacterial activities in Huang-huai sheep.

### Slaughter performance

Slaughter performance, as indicated by carcass weight, slaughter ratio, net meat weight, and eye muscle area, is crucial for assessing the production performance and determining the amount of saleable meat in animals, ultimately impacting sheep farmers’ income [5]. Moreover, an increased LEA value indicates higher meat production performance of sheep [5]. Wang et al [7,8] showed that time-limited grazing combined with supplementation increased carcass weight and DP. The present experiment showed that lambs in the ITGP group exhibited increased DP and an upward trend in HCW (p = 0.10) than those in CON group. The higher efficiency of grazing activities, combined with the nutritional benefits obtained from fresh herbages in the limited grazing time, likely contribute to the higher LEA values in sheep of the ITGP group. Furthermore, internal organ index reflects to some extent growth, development, and body function of animals. Wang et al [7] reported that restricted grazing time combined with supplementation did not affect animal joints except for a part of the breast. In the present study, non-carcass components of Huang-huai sheep were not affected by the feeding regimens, except for empty gastrointestinal tract, which may be explained the fact that the intake of diverse herbage compounds in grazing pasture by sheep increase in the percentage of the digestive tract, including a heavier small intestine and a higher rumen volume, as well as higher SLW in animals of the ITGP group.

### Edible quality attributes

The pH value is a crucial factor affecting water-holding capacity and tenderness of meat. An increase in pH value indicates higher fresh-keeping performance of meat [18]. In addition, DL value reflects the water-holding capacity of muscle [5], and a low value indicates high meat juiciness. In this study, no significant differences were observed among three feeding regions for pH and DL value of BB muscle. Wang et al [7], Önenç et al [19] and da Silva et al [20] also found that no differences on pH and juiciness of meat of sheep submitted to restricted grazing time combined with supplementation or to grazing and supplementation compared to indoor-fed animals. This also suggests that indoor feeding combined with time-limited grazing or a grazing system did not alter fresh-keeping performance and juiciness of meat.

Meat color is an important attribute influencing the appearance and economic value of meat, which often affects consumers’ purchase decisions [3]. The study by Wang et al [7] found that meat from animals submitted to time-limited grazing on grassland combined with supplementation decreased b* value, and similar L* and a* values compared to those submitted to indoor feeding. Önenç et al [19] and Majdoub-Mathlouthi et al [21] described that supplemented and grazing sheep exhibited similar meat L*, a*, and b* values compared to indoor-fed animals. The results of this study showed that lambs in the color of BB muscle in lambs of the IEGP group did not change compared to that in indoor-fed animals. In contrast, the BB muscle of lambs in the ITGP group showed decreased L* value, which could be possibly due to the different levels of physical activity to which animals in the ITGP group were submitted [22]. However, lambs in all feeding regimens evaluated in this study exhibited L* values >34 and a* values >9.5, which are considered above the level acceptable by consumers [23]. Therefore, the three feeding systems produced lamb that meet consumers’ demand in terms of meat color, although no significant difference was found in pH, DL, and L*, a*, and b* values.

### Chemical composition attributes

Muscle composition, including moisture, CP, IMF, and ash, are among the main attributes of the meat quality in small ruminants [7]. In this study, moisture, CP and IMF contents in meat from animals submitted to the feeding regions proposed herein were found within the ranges of 70.8 to 80.3 g/100 g, 18.5 to 23.4 g/100 g, and below 5.0 g/100 g, respectively, which are indicative of good quality and lean meat [24]. Previous reports demonstrate that the content of moisture, IMF, and ash in *Longissimus lumborum* of lambs raised under time-limited grazing on grassland combined with supplementary indoor feeding were comparable to those of animals raised on indoor feeding [7,8], but with higher CP content [7]. The present experiment also indicated that the BB muscle of Huang-huai sheep in ITGP group had increased CP content, which could partly explain the higher nutritional quality of the resulting meat. This increase in results may be because of a higher CP degradation and an increased CP turnover in the muscle [25]. However, further research is needed to elucidate the underlying mechanisms of how different feeding regimens affect protein content in Huang-huai sheep meat.

### Fatty acid composition attributes

The FA composition and content are closely correlated with meat quality and human health [2,21]. Most SFAs are considered potentially hypercholesterolemic FA and, as such, increase cholesterol production, thus promoting the accumulation of low-density lipoprotein, which in turn leads to an increased risk of developing cardiovascular diseases [2,21]. However, stearic acid (C18:0) is considered neutral and not associated with an increased risk for cardiovascular diseases [26]. In this study, C17:0 was found significantly higher in animals in the ITGP group compared to those in the IEGP group. However, the contents of C18:0 as well as the other SFAs and total SFA were not affected by the proposed feeding systems, thus indicating that restricted grazing or combining grazing time with adding levels of feeding diets had no adverse effects on SFA accumulation in lambs. In contrast, MUFA and PUFA are considered hypocholesterolemic FAs. In this study, the BB muscle of animals in the ITGP group had significantly increased contents of C18:2n6, C20:4n6, C20:3n6, and total PUFA, while that of animals in the IEGP group had significantly increased levels of C22:1n-9, C18:2n6, C20:4n6, and total PUFA. This indicates that combining grazing with indoor feeding could potentially promote accumulation of MUFA and PUFA in sheep meat, which can be thus considered healthy for humans. Conversely, no differences were found in the levels of eicosapentaenoic (EPA, C20:5n-3) and docosahexaenoic (DHA, C22:6n-3) acids among the three feeding systems. Nevertheless, 100 g of meat from all three feeding systems contained 130 mg of EPA+DHA, which meets 52% of the recommended daily intake (250 mg/d EPA+DHA) for healthy individuals [27].

From a nutritional standpoint, a recommended dietary ratio of PUFA/SFA for a healthy diet is >0.4 [28]. In the present study, both the IEGP and ITGP groups (average values of 0.54 and 0.58, respectively) had significantly higher PUFA/SFA ratios compared to the CON group (0.35), thus indicating a more favorable FA composition. The ratio of n-6/n-3 PUFA up to 4 is recommended in human diet to help maintain good cardiovascular health [29]. In this study, the n-6/n-3 PUFA ratio in Huang-huai sheep meat, which should ideally be around 4 for maintaining cardiovascular health, did not differ significantly among the three feeding regimens and was higher than the recommended value. Gruffat et al [29] discovered that the n-6/n-3 PUFA ratio in lamb meat obtained from Romane breed reared under different feeding regimens was 5.9, 1.8, and 1.6, respectively. Therefore, the nutritive value of FA is more pronounced in Huang-huai sheep compared to other sheep breeds. Moreover, a lower C16:0/C18:1 ratio is beneficial and improves insulin sensitivity, since it protects against risk of developing diabetes in humans [2]. Interestingly, lambs of the IEGP and ITGP groups exhibited a significantly increased C16:0/C18:1 ratio compared to those in the CONT group, which is similar to the results obtained with Tan lambs raised grazing on pasture and indoor feeding [2]. These findings indicate that, although most individual FA contents in the muscles of Huang-huai sheep were not significantly affected by feeding regimens, the nutritional value of the meat of animals under time-limited grazing combined with supplementation or grazing and supplementation was more favorable. These findings provide a novel discovery that could enhance market acceptance of Huang-huai sheep raised in feedlots. However, further research is required to elucidate the mechanisms underlying the improved FA deposition in lambs submitted to the three different feeding regimens proposed herein.

### Economic efficiency

The pursuit of economic benefit serves as the important objective for all sheep enterprises or farmers, playing a crucial role in the advancement of the sheep industry. The present experiment showed that economic efficiency of lambs was most favorable in ITGP group compared to the CON and IEGP groups, despite not having the lowest daily feed cost. Consequently, it suggests that substitution of indoor feeding or supplemented grazing by time-limited grazing combined with supplementation may be more profitable to the fattening period of lambs. In addition, it is necessary to further examine the potential effects of the feeding system on the flavor attributes and underlying mechanisms of sheep meat.

## CONCLUSION

The present study investigated the effects of different feeding regimens on various aspects of Huang-huai sheep meat, including serum antioxidant and immunity capacities, slaughter performance, and FA composition. The findings demonstrated that combining indoor feeding with grazing methods, such as time-restricted or eight-hour daily grazing, resulted in higher antioxidant and immunity capacities and superior quality and nutritional attributes to Huang-huai sheep meat compared to indoor-fed animals. Moreover, lambs receiving indoor feeding and with an 8-h daily access to pasture exhibited similar carcass traits compared to indoor-fed only animals. However, carcass traits of lambs receiving indoor feeding and with an eight-hour daily access to pasture were inferior to those of indoor-fed only animals allowed time-restricted grazing on pasture. Future research is needed to elucidate the mechanisms underlying the effects of indoor feeding combined with time-restricted pasture grazing on carcass traits and meat FA composition of Huang-huai sheep. In summary, differences observed among feeding systems in terms of performance revealed that indoor feeding combined with time-restricted grazing pasture can be potentially considered as a more effective grazing management for Huang-huai sheep.

## Figures and Tables

**Table 1:** Ingredients and chemical composition of indoor supplementary feeding diet (dry-matter basis)

Items	Indoor supplementary feeding diet	Grazing pasture
Ingredients (%)		
Whole corn silage	45.0	
Corn grain	30.2	
Soybean meal	14.0	
Wheat bran	7.8	
NaHCO_3_	1.0	
NaCl	1.0	
Premix^1)^	1.0	
Total	100	
Chemical composition (%)		
Dry matter	46.8	17.3
Crude protein	16.4	12.3
Neutral detergent fiber	30.3	14.0
Acid detergent fiber	18.3	65.1
Crude ash	8.3	39.3
Calcium	0.7	
Phosphorus	0.4	

1) Each 1 kg premix contained 3,500 IU vitamin A, 1,000 IU vitamin D, 40 IU vitamin E, 50 mg iron, 40 mg manganese, 10 mg copper, 40 mg Zinc, 0.3 mg selenium.

**Table 2:** Effects of different feeding systems on the plasma antioxidant and immunity parameters of Huang-huai lambs

Items	Treatment^1)^			SEM	p-value

	CON	IEGP	ITGP		
Glutathione peroxidase (pg/mL)	1,127.25^b^	1,471.18^a^	1,524.80^a^	89.78	<0.001
Immunoglobulin A (μg/mL)	113.94^b^	178.28^a^	166.80^a^	11.71	<0.001
Immunoglobulin G (mg/mL)	15.94^b^	37.86^a^	30.68^a^	2.96	<0.001
Immunoglobulin M (μg/mL)	1,020.48^b^	1,710.72^a^	1,556.99^a^	109.88	<0.001

SEM, standard error of the mean.

1) CONT, indoor feeding with no access to pasture; IEGP, indoor feeding combined with 8-h per day access to artificial grazing pasture; ITGP, indoor feeding combined with time-limited (4 h) daily access to grazing on artificial pasture.

a,b Means within a row with different subscripts differ when p<0.05.

**Table 3:** Effects of different feeding systems on the slaughter performance of Huang-huai lambs

Items	Treatment^1)^			SEM	p-value

	CON	IEGP	ITGP		
Dry matter intake (kg/d)	1.31^a^	0.86^b^	1.24^a^	0.07	<0.001
Carcass traits					
SLW (kg)	48.78^ab^	41.88^b^	53.16^a^	1.82	0.026
Hot carcass weight (kg)	23.73^ab^	20.50^b^	27.83^a^	1.06	0.008
Dressing percentage (%)	48.67^b^	48.87^b^	52.36^a^	0.72	0.053
Meat weight (kg)	17.85^ab^	14.01^b^	20.97^a^	1.04	0.012
Meat yield (%)	36.24^ab^	33.36^b^	39.52^a^	1.14	0.078
Bone weight (kg)	5.76	5.29	6.74	0.31	0.143
Bone yield (%)	11.84	12.62	12.85	0.53	0.742
Meat/bone	3.08	2.74	3.19	0.14	0.419
Grade rule (mm)	4.51	3.42	4.88	0.33	0.172
Loin eye area (cm^2^)	19.58^b^	19.31^b^	25.68^a^	1.07	0.013
Non-carcass components, (%) SLW					
Heart (%)	0.39	0.44	0.41	0.01	0.257
Liver (%)	1.98	2.26	1.80	0.11	0.258
Spleen (%)	0.47	0.33	0.21	0.06	0.258
Lungs (%)	0.97	1.10	1.02	0.05	0.576
Kidney (%)	0.28	0.31	0.27	0.01	0.269
pancreas (%)	0.09	0.10	0.11	0.01	0.448
Empty gastrointestinal tract (%)	5.97^b^	6.66^a^	6.62^a^	0.14	0.050
Body fat deposition, (%) HCW					
Subcutaneous fat (%)	4.72^a^	2.82^b^	3.41^b^	0.30	0.016
Omental and mesenteric fat (%)	4.19	2.90	3.13	0.40	0.401
Kidney fat (%)	1.00	0.56	0.79	0.10	0.202
Tail fat (%)	0.50	0.75	0.78	0.09	0.348

SEM, standard error of the mean; SLW, slaughter live weight; HCW, hot carcass weight.

1) CONT, indoor feeding with no access to pasture; IEGP, indoor feeding combined with 8-h per day access to artificial grazing pasture; ITGP, indoor feeding combined with time-limited (4 h) daily access to grazing on artificial pasture.

a,b Means within a row with different subscripts differ when p<0.05.

**Table 4:** Effects of different feeding systems on the edible quality attributes in the biceps brachii muscle of Huang-huai lambs

Items	Treatment^1)^			SEM	p-value

	CON	IEGP	ITGP		
pH_45 min_	6.46	6.44	6.56	0.07	0.765
pH_24 h_	6.20	6.10	6.25	0.06	0.656
Lightness	44.54^a^	35.47^b^	42.68^a^	1.45	0.021
Redness	19.39	19.97	16.97	0.91	0.402
Yellowness	14.17	14.41	12.81	0.65	0.599
Chroma	24.17	24.81	21.33	0.94	0.315
Hue angle	36.92	35.37	37.41	1.45	0.859
Drip loss (%)	2.15	1.96	2.24	0.11	0.600

SEM, standard error of the mean.

1) CONT, indoor feeding with no access to pasture; IEGP, indoor feeding combined with 8-h per day access to artificial grazing pasture; ITGP, indoor feeding combined with time-limited (4 h) daily access to grazing on artificial pasture.

a,b Means within a row with different subscripts differ when p<0.05.

**Table 5:** Effects of different feeding systems on the fatty acid composition (mg/100 g of meat) in the biceps brachii muscle of Huang-huai lambs

Items	Treatment^1)^			SEM	p-value

	CON	IEGP	ITGP		
SFA					
C10:0	12.86	15.39	18.08	1.47	0.484
C11:0	147.26	168.41	146.13	14.29	0.805
C12:0	21.13	20.18	15.31	2.06	0.544
C14:0	119.19	92.36	119.29	10.74	0.547
C15:0	19.79	18.90	22.21	1.75	0.762
C16:0	1328.78	984.47	1,096.98	83.53	0.262
C17:0	65.21^ab^	52.29^b^	94.54^a^	6.79	0.029
C18:0	1057.65	1057.64	1,087.78	53.19	0.971
C20:0	17.21	14.00	14.86	1.05	0.481
C21:0	13.39	10.03	10.05	0.94	0.363
MUFA					
C14:1	7.88	13.68	16.32	2.03	0.527
C16:1	99.96	101.60	123.76	7.85	0.431
C17:1	45.84	40.97	66.55	5.57	0.154
C20:1	11.90	10.79	13.82	0.93	0.455
C18:1n9	1,062.70	1,054.69	1,226.06	65.56	0.532
C22:1n-9	33.39^b^	60.14^a^	49.40^ab^	3.93	0.014
PUFA					
C18:3n6	24.12	22.99	21.82	1.54	0.868
C18:2n6	488.28^b^	686.47^a^	845.40^a^	48.56	0.005
C20:4n6	255.59^b^	377.83^a^	362.97^a^	19.56	0.015
C20:5n3	88.79	110.51	113.11	5.69	0.181
C20:3n6	35.06^b^	41.11^ab^	48.58^a^	2.48	0.088
C20:2	11.53^ab^	9.73^b^	17.94^a^	1.53	0.074
C22:6n3	25.74	27.66	26.70	1.12	0.833
C22:2	35.82	35.29	36.68	2.56	0.979
SFA	2,783.42	2,412.58	2,612.16	133.58	0.573
MUFA	1,257.73	1,275.03	1,490.47	69.29	0.353
PUFA	960.64^b^	1,311.60^a^	1,465.11^a^	67.91	0.003
n-3 PUFA	110.24	138.17	135.36	7.00	0.228
n-6 PUFA	767.99^b^	1,087.30^a^	1,226.55^a^	60.76	0.002
n-6/n-3 PUFA	7.86	7.95	9.16	0.51	0.559
PUFA/SFA	0.35^b^	0.55^a^	0.58^a^	0.03	0.003
C16:0/C18:1	1.24^a^	0.96^b^	0.90^b^	0.06	0.025

SEM, standard error of the mean; SFA, saturated fatty acids; MUFA, monounsaturated fatty ac-ids; PUFA, polyunsaturated fatty acids.

1) CONT, indoor feeding with no access to pasture; IEGP, indoor feeding combined with 8-h per day access to artificial grazing pasture; ITGP, indoor feeding combined with time-limited (4 h) daily access to grazing on artificial pasture.

a,b Means within a row with different subscripts differ when p<0.05.

**Table 6:** Effects of different feeding systems on the economic efficiency of Huang-huai lambs

Items	Treatment^1)^		

	CON	IEGP	ITGP
Sheep purchase price (Dollars/head)	150.53	153.65	146.74
Total feed cost (Dollars/112 d/head)	44.67	55.31	55.27
Concentrate	23.53	15.45	22.27
Whole corn silage	21.14	13.88	20.01
Rental fees for grazing grassland (Dollars/112 d/head)	0.00	23.39	11.69
Daily feed cost (Dollars/d/head)	0.40	0.49	0.49
Carcass price (Dollars/head)	246.30	212.77	288.95
Economic efficiency	51.10	3.81	86.94

1) CONT, indoor feeding with no access to pasture; IEGP, indoor feeding combined with 8-h per day access to artificial grazing pasture; ITGP, indoor feeding combined with time-limited (4 h) daily access to grazing on artificial pasture.

## References

[1] Zhao J, Quan K, Zhang Z (2021). Mutton performance of Huang-huai sheep. J Inner Mongolia Agric Univ.

[2] Wang B, Wang YJ, Zuo SX (2021). Untargeted and targeted metabolomics profiling of muscle reveals enhanced meat quality in artificial pasture grazing tan lambs via rescheduling the rumen bacterial community. J Agric Food Chem.

[3] Zhang Z, Wang X, Jin Y, Zhao K, Duan Z (2022). Comparison and analysis on sheep meat quality and flavor under pasture-based fattening contrast to intensive pasture-based feeding system. Anim Biosci.

[4] Priolo A, Micol D, Agabriel J (2001). Effects of grass feeding systems on ruminant meat colour and flavour. A review. Anim Res.

[5] Su Y, Sun X, Zhao S (2022). Dietary alfalfa powder supplementation improves growth and development, body health, and meat quality of Tibetan sheep. Food Chem.

[6] Valentini J, da Silva AS, Fortuoso BF (2020). Chemical composition, lipid peroxidation, and fatty acid profile in meat of broilers fed with glycerol monolaurate additive. Food Chem.

[7] Wang B, Wang Z, Chen Y (2021). Carcass traits, meat quality, and volatile compounds of lamb meat from different restricted grazing time and indoor supplementary feeding systems. Foods.

[8] Wang Z, Chen Y, Luo H (2015). Influence of restricted grazing time systems on productive performance and fatty acid composition of longissimus dorsi in growing lambs. Asian-Australas J Anim.

[9] Valadez-Garcia KM, Avendano-Reyes L, Diaz-Molina R (2021). Free ferulic acid supplementation of heat-stressed hair ewe lambs: Oxidative status, feedlot performance, carcass traits and meat quality. Meat Sci.

[10] Payne CE, Pannier L, Anderson F (2020). Lamb age has little impact on eating quality. Foods.

[11] Tuell JR, Kim HW, Zhang J (2021). Arginine supplementation may improve color and redox stability of beef loins through delayed onset of mitochondrial-mediated apoptotic processes. Food Chem.

[12] Grochowska E, Borys B, Lisiak D, Mroczkowski S (2019). Genotypic and allelic effects of the myostatin gene (MSTN) on carcass, meat quality, and biometric traits in Colored Polish Merino sheep. Meat Sci.

[13] Latimer GW, AOAC International (2012). Official methods of analysis of AOAC International.

[14] Chen X, Zhao N, Zhang Y, Geng Z (2017). The fatty acid in muscles and expression of PPARα, FADS2 and ME1 genes in liver of chinese Wanxi white geese in fattening period. Acta Vet Zootech Sin.

[15] Chen Y, Gong X, Yang T, Ginkgo Biloba L (2021). Residues partially replacing alfalfa hay pellet in pelleted total mixed ration on growth performance, serum biochemical parameters, rumen fermentation, immune function and meat quality in finishing Haimen white goats. Animals.

[16] Qi K, Men X, Wu J (2023). Effects of feeding methods on carcass traits, blood indexes and meat quality of “Lvjiahei” black pig China. Chinese J Anim Sci.

[17] Hu B, Wan W, Gong Y (2018). Effects of feeding models on slaughter performance, serum biochemical indexes and intestinal morphology of different strains of Jingyang chicken. China Poult.

[18] Zhang C, Luo J, Yu B (2015). Dietary resveratrol supplementation improves meat quality of finishing pigs through changing muscle fiber characteristics and antioxidative status. Meat Sci.

[19] Onenc S, Ozdogan M, Aktumsek A (2015). Meat quality and fatty acid composition of Chios male lambs fed under traditional and intensive conditions. Emir J Food Agric.

[20] da Silva PCG, Itavo CCBF, Itavo LCV (2020). Carcass traits and meat quality of Texel lambs raised in Brachiaria pasture and feedlot systems. Anim Sci J.

[21] Majdoub-Mathlouthi L, Said B, Kraiem K (2015). Carcass traits and meat fatty acid composition of Barbarine lambs reared on rangelands or indoors on hay and concentrate. Animal.

[22] Caneque V, Velasco S, Diaz MT, De Huidobrob FR, Pérezc C, Lauzurica S (2003). Use of whole barley with a protein supplement to fatten lambs under different management systems and its effect on meat and carcass quality. Anim Res.

[23] Khliji S, van de Ven R, Lamb TA, Lanza M, Hopkins DL (2010). Relationship between consumer ranking of lamb colour and objective measures of colour. Meat Sci.

[24] Bezerra LS, Barbosa AM, Carvalho GGP (2016). Meat quality of lambs fed diets with peanut cake. Meat Sci.

[25] Millward DJ, Waterlow JC (1978). Effect of nutrition on protein turnover in skeletal muscle. Feder Proc.

[26] Abdallah A, Zhang P, Elemba E (2020). Carcass characteristics, meat quality, and functional compound deposition in sheep fed diets supplemented with Astragalus membranaceus by-product. Anim Feed Sci Technol.

[27] EFSA (2010). Scientific opinion on dietary reference values for fats, including saturated fatty acids, polyunsaturated fatty acids, monounsaturated fatty acids, trans fatty acids, and cholesterol. EFSA J.

[28] Wood JD, Richardson RI, Nute GR (2003). Effects of fatty acids on meat quality: a review. Meat Sci.

[29] Gruffat D, Durand D, Rivaroli D, do Prado IN, Prache S (2020). Comparison of muscle fatty acid composition and lipid stability in lambs stall-fed or pasture-fed alfalfa with or without sainfoin pellet supplementation. Animal.

